# Detection of a Second KAP22 Family Member in Sheep and Analysis of Its Genetic Variation and Associations with Selected Wool Fibre Traits

**DOI:** 10.3390/ani15192770

**Published:** 2025-09-23

**Authors:** Lingrong Bai, Huitong Zhou, Jinzhong Tao, Jon G. H. Hickford

**Affiliations:** 1 International Wool Research Institute, Faculty of Animal Science and Technology, Gansu Agricultural University, Lanzhou 730070, China; lingrong.bai@lincolnuni.ac.nz (L.B.); huitong.zhou@lincoln.ac.nz (H.Z.); tao_jz@nxu.edu.cn (J.T.); 2 Gene-Marker Laboratory, Faculty of Agriculture and Life Sciences, Lincoln University, Lincoln 7647, New Zealand; 3 College of Animal Science and Technology, Ningxia University, Yinchuan 750021, China

**Keywords:** keratin-associated protein, KAP22-2, variation, association, wool traits

## Abstract

**Simple Summary:**

Wool quality depends in part on the proteins that make up the fibres, including a class of proteins known as the keratin-associated proteins (KAPs). The gene *KRTAP22-2*, which encodes a high glycine-tyrosine KAP, was recently identified in goats and reported to affect the important cashmere fibre trait of mean fibre diameter. It has not been identified in other species, including sheep and humans. In this study, we identified ovine *KRTAP22-2* and revealed two sequence variants of the gene. We investigated whether different genotypes were associated with wool traits in Chinese Tan lambs, but found no association between them and the wool traits examined. We searched genetic databases to see if the gene is expressed in sheep, but did not find any mRNA matches. These findings suggest potential species-specific differences and emphasise the need for further research to better understand this gene’s role in sheep.

**Abstract:**

The keratin-associated proteins (KAPs) are a class of wool proteins. They form a matrix that cross-links the wool intermediate filament keratins. The KAPs are thought to affect wool fibre structure and properties and have been associated with variation in wool fibre traits. There are many KAP genes in sheep, but not all have been identified. Recently a second member of the KAP22 gene family, *KRTAP22-2*, was identified in goats, and variation in this caprine gene was associated with cashmere fibre traits. In this study, we identified ovine *KRTAP22-2*. To ascertain the extent of variation in *KRTAP22-2,* sheep from eight breeds were investigated using polymerase chain reaction (PCR) followed by single-strand conformational polymorphism (SSCP) analysis. This revealed two unique banding patterns, which upon sequencing gave two novel DNA sequences. These differed by two single nucleotide polymorphisms in the coding region. Three genotypes of the novel *KRTAP22-2* sequences were observed in the eight sheep breeds studied. The ovine *KRTAP22-2* variant sequences were similar to a goat *KRTAP22-2* variant, but a search of ovine expressed sequence tags revealed no matching mRNA sequences in the ovine databases. In a second part of the study, no association was found between the *KRTAP22-2* genotypes and mean fibre diameter, fibre diameter standard deviation, coefficient of variation in fibre diameter, and mean fibre curvature, for either the fine wool or heterotypic hair fibres of 255 Chinese Tan lambs. These results suggests that sheep have a *KRTAP22-2* gene, but that there may be species-specific differences in the gene’s expression or function. The gene may not affect wool traits in the way that it appears to in goats.

## 1. Introduction

Wool is a natural fibre renowned for its unique properties. This makes it valuable in a wide range of applications, for which it has been described as “nature’s wonder fibre” [1], and it is a sustainable fibre choice [2]. A challenge for the wool industry is the naturally occurring variation in wool fibre characteristics. This can limit its use and reduce its value.

The unique properties of wool fibres are attributed to their complex structure [3]. They are composed of a large number of different proteins that are arranged into a complex structure. While the mechanisms by which these proteins are assembled and organised remain unclear, the sequences, content, and spatial location of individual proteins are becoming known.

Wool fibres are principally composed of two classes of proteins, wool keratins and keratin-associated proteins (KAPs). The KAPs covalently cross-link the keratins to create a matrix. In sheep, 17 wool keratins (10 type I and 7 type II) have been described [4,5,6,7], a number that is comparable to what is detected in humans [8]. However, the KAPs are more diverse, with a recent analysis of sheep genome sequence suggesting 102 KAP genes (designated as *KRTAPs*), including 23 that belong to the high-sulphur (HS) group, 48 that belong to the ultra-high-sulphur (UHS) group, and 31 that belong to the high-glycine-tyrosine (HGT) group [9]. This is more than reported in humans [10,11,12]. The *KRTAP* genes of sheep are widely reported to affect wool traits and this has implications for wool quality and breeding [9].

The greater number of *KRTAP* genes in sheep appears to be due to them having an expanded repertoire of genes in the HGT group. For example, while the human KAP6 family contains only three genes [11], five genes are present in sheep [13]. The HGT-KAPs are of particular interest with wool because they are preferentially expressed in the orthocortex of the fibre, and their presence varies both between and within breeds and species [14]. They account for less than 3% of the total protein content in human hair and wool from the Lincoln breed of sheep, but range from 4% to 12% in Merino wool [15]. The felting lustre mutant phenotype in Merino sheep is associated with having a lower HGT-KAP content [16] and together these findings suggest a role for the HGT-KAPs in determining wool fibre characteristics.

One HGT-KAP family has been called KAP22. Humans are reported to have a single gene, *KRTAP22-1* [11], whereas goats possess two genes, including the recently identified *KRTAP22-2* [17]. This study reported that variation in caprine *KRTAP22-2* was associated with differences in the valuable trait of cashmere mean fibre diameter (MFD).

Given these observations, the following study was undertaken to ascertain whether an ovine analogue for caprine *KRTAP22-2* exists and whether the sheep gene is variable. Having established that the gene existed and that sequence variation occurred in it, we investigated whether variation in the gene was associated with wool traits in Chinese Tan sheep, a breed renowned for producing wool with a distinctive ‘spring-like’ crimp in early life [18].

## 2. Materials and Methods

### 2.1. Sheep and Wool Trait Measurement

This research used two groups of sheep that were analysed in separate parts of the study. The first group included 49 mixed-age sheep, randomly selected from seven farms that had different breeds, including Merino (*n* = 13), Corriedale (*n* = 11), New Zealand Romney (*n* = 9), Texel (*n* = 6), South Suffolk (*n* = 5), Poll Dorset (*n* = 3), and Coopworth (*n* = 2). These sheep were studied solely for the purpose of detecting *KRTAP22-2* and ascertaining whether genetic variation existed in the gene. Neither wool samples nor corresponding wool trait data were available for them.

The second group comprised 255 lambs (*n* = 115 males and *n* = 140 females) from the Chinese Tan sheep breed, the recorded offspring of 11 rams. These were maintained in a single flock until sampling. Wool samples were collected using shears from the mid-side region of these lambs at “er-mao” (day 35 post-partum). The two types of fibres in the samples, fine wool fibres and heterotypic hair fibres, were manually separated based on their respective differences in length and diameter. The separation involved taking staples of the wool and pressing the fibre bases (that are closest to the skin) onto a board covered in cotton flannel and selectively pulling out the longer, coarser heterotypic hair fibres by hand. This procedure was repeated until all the heterotypic hair fibres were removed from the finer wool.

For both fibre types, the following wool traits were measured: MFD, fibre diameter standard deviation (FDSD), coefficient of variation in fibre diameter (CVFD), and mean fibre curvature (MFC). The fine wool fibres were analysed by Pastoral Measurements Limited (Timaru, New Zealand), and the heterotypic hair fibres were tested at the New Zealand Wool Testing Authority (Napier, New Zealand) using standardised wool testing methods developed by the International Wool Textile Organisation.

Blood samples were collected from the sheep by nicking the ear tip and collecting the blood on TFN paper (Munktell Filter AB, Falun, Sweden). These were dried and stored in the dark until required. Genomic DNA for PCR amplification was purified by incubating 1.2 mm diameter punches of the blood on the card in 20 mM NaOH solution for 30 min at room temperature. Following aspiration of the NaOH solution and a subsequent single equilibration wash with 1× TE^−1^ buffer (10 mM Tris-HCl, 0.1 mM EDTA, pH 8.0), the samples as punches were air dried and stored until required.

### 2.2. PCR Amplification and Single-Strand Conformational Polymorphism Analysis

To identify potential analogues of the caprine *KRTAP22-2* gene, a BLAST search was performed using the caprine *KRTAP22-2 A*, *B*, *C*, and *D* allele sequences (GenBank accession numbers MF143986-MF143988 and *D* sequence derived from Chen et al. [17], respectively) against the sheep genome assembly ARS-UI_Ramb_3.0 (GCF_016772045.2). The ovine sequence with the greatest similarity to the caprine sequences was presumed to be ovine *KRTAP22-2*. This region of the ovine genome sequence was then used to design the primers 5′-TGAGTCTAGCAGTGCCTGTG-3′ (forward) and 5′-GATCCTCATAAAAGAACATCC-3′ (reverse) for PCR amplification of the putative ovine *KRTAP22-2*. These were made by Integrated DNA Technologies (Coralville, IA, USA).

The PCR amplifications were carried out in 15-μL reactions, each containing a single washed TFN paper punch, plus 150 μM of each dNTP (Bioline, London, UK), 0.25 μM of each primer, 2.5 mM Mg^2+^, 0.5 U of *Taq* DNA polymerase (Qiagen, Hilden, Germany), and 1× the reaction buffer that was supplied with the *Taq* enzyme. PCR amplification was undertaken in S1000 thermal cyclers (Bio-Rad, Hercules, CA, USA), starting with a 2-min denaturation at 94 °C, followed by 35 cycles of denaturation at 94 °C for 30 s, annealing at 58 °C for 30 s, and elongation at 72 °C for 30 s. A final elongation at 72 °C for 5 min completed the process.

To screen for sequence variation, the PCR products were subjected to single-strand conformational polymorphism (SSCP) analysis. For each sample, a 0.7 μL aliquot of PCR product was mixed with 7 μL of gel loading dye (0.025% bromophenol blue, 0.025% xylene-cyanol, 98% formamide, 10 mM EDTA). This was incubated at 95 °C for 5 min to denature the DNA strands, then rapidly cooled on wet ice to minimise the likelihood of renaturation. The samples were then rapidly loaded on 16 cm × 18 cm, 14% acrylamide–bisacrylamide (37.5:1) (Bio-Rad) gels that contain 4% glycerol. Electrophoresis was carried out at 390 volts and 14 °C for 19 h in a 0.5× TBE running buffer in Protean II xi cells (Bio-Rad). Following electrophoresis, the gels were immersed in a solution of 10% ethanol, 0.5% acetic acid, and 0.2% silver nitrate for 10 min, briefly rinsed with distilled water, and developed in 3% NaOH with 0.1% formaldehyde. Once bands became visible against the yellow background, the development was stopped by removing the developing solution and the addition of a solution containing 10% ethanol and 0.5% acetic acid.

### 2.3. DNA Sequencing and Sequence Analysis

The PCR products representative of different SSCP banding patterns from sheep that appeared to be homozygous at the target locus were Sanger sequenced in triplicate in both directions using the service provided by the Lincoln University DNA sequencing facility (Lincoln University, New Zealand). The sequence chromatograms were checked to confirm the presence of single nucleotide polymorphisms (SNPs), and subsequent sequence alignments and translations were undertaken using DNAMAN XL (version 10, Lynnon BioSoft, San Ramon, CA, USA).

BLAST searches (megablast, organism: sheep) against the ovine expressed sequence tag (EST) database were performed using the NCBI BLAST tool available at https://blast.ncbi.nlm.nih.gov/Blast.cgi (accessed on 20 August 2025). The Query Sequence was the entire coding region of the *KRATP22-2* sequence.

### 2.4. Statistical Analyses

Statistical analyses were conducted using Minitab (version 16, Minitab Inc., State College, PA, USA). General linear models were used to ascertain if there was an association between the *KRTAP22-2* genotypes and wool traits. Only genotypes with a frequency greater than 5% were included in the analysis. To control for the increased risk of type I error due undertaking multiple comparisons, a Bonferroni correction was applied. The models accounted for both sire and lamb sex effects, as both factors were found to influence the wool traits. The model used was as follows: Y_jkl_ = µ + G_j_ + S_k_ + P_l_ + e_jkl_, where Y_jkl_ represent the observed trait value for the jkl^th^ animal, µ is the overall mean, G_j_ is the fixed effect of the j^th^ genotype, S_k_ is the effect of lamb sex, P_l_ is the effect of the l^th^ sire, and e_jkl_ is the residual error.

## 3. Results

**Figure 1:**
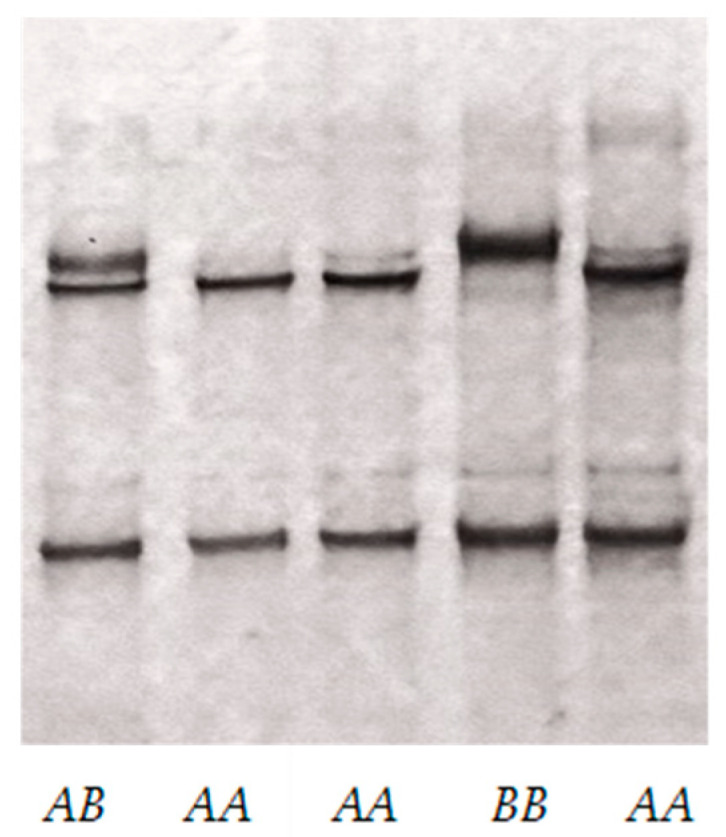
PCR-SSCP gel banding patterns for the two ovine *KRTAP22-2* variant sequences. Two different banding patterns (*A* and *B*), corresponding to two different DNA sequence variants are observed in both heterozygous and homozygous sheep.

**Figure 2:**
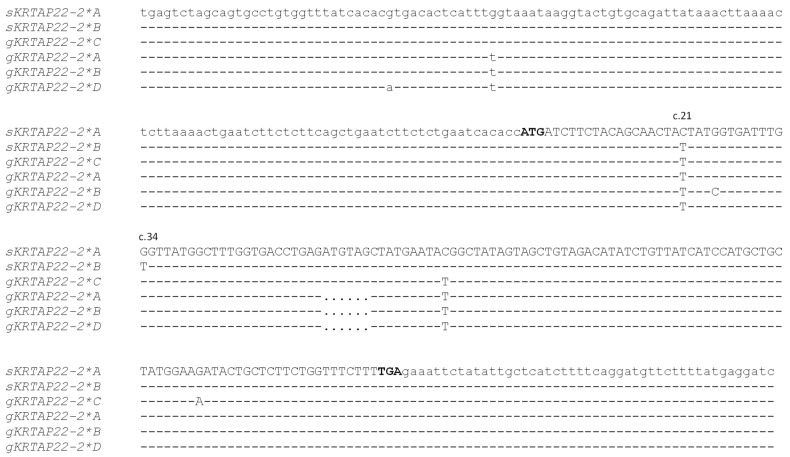
Nucleotide sequence alignment of the two ovine *KRTAP22-2* variants (marked as *sKRTAP22-2*) and four caprine *KRTAP22-2* variants (marked as *gKRTAP22-2*). Nucleotides of the coding region are shown in uppercase letters. The putative start and stop codons are indicated in bold. Nucleotides identical to those in the top sequence are represented by dashes, and dots indicate missing nucleotides. The positions of the two SNPs identified between the ovine variants are marked above the sequences.

The PCR amplification produced a single fragment of the expected size (323 bp) in all sheep samples. This size was consistent with predictions based on the ovine reference genome sequence. Analysis of the PCR products by SSCP revealed two distinct banding patterns (Figure 1) and Sanger sequencing of PCR amplicons representative of apparently homozygous SSCP patterns revealed two nucleotide sequences (named variant *A* and variant *B*; Figure 2). These variants differed by two SNPs: c.21C/T and c.34G/T. The latter would lead to an amino acid change (p.Gly12Cys) if the gene was expressed. Variant *A* was identical to the sequence found on chromosome 1 (NC_056054.1) of the ovine reference genome assembly ARS-UI_Ramb_v3.0 (GCF_016772045.2).

Alignment of the putative ovine *KRTAP22-2* sequences with the caprine *KRTAP22-2* variant sequences revealed that both ovine variants were most similar to goat variant *C* (Figure 2). A search of the ovine EST databases did not identify any transcripts matching either variant *A* or *B*, returning the result “no significant similarity found”, which suggests that no corresponding expression data are currently available.

Three genotypes (*AA*, *AB*, and *BB*) were observed in the 49 sheep screened for genetic variation as follows: Merino (11× *AA* and 2× *AB*), Corriedale (11× *AA*), New Zealand Romney (7× *AA* and 2× *AB*), Texel (5× *AA* and 1× *BB*), South Suffolk (4× *AA* and 1× *AB*), Poll Dorset (2× *AA* and 1× *AB*), and Coopworth (2× *AA*). This gives overall genotype frequencies of 85.7% *AA*, 12.3% *AB*, and 2% *BB*, respectively.

In the group of 255 Tan sheep used for the association analysis, the same genotypes were detected with frequencies of 78.0% (*AA*), 21.2% (*AB*), and 0.8% (*BB*). Due to the low frequency of the *BB* genotype, association analysis between genotypes and wool traits was limited to the *AA* and *AB* genotypes. No significant differences were found between these two genotypes for MFD, FDSD, CVFD, or MFC for either the fine wool fibres or the heterotypic fibres (Table 1); however, a tendency towards significance (*p* = 0.074) was observed for the CVFD of heterotypic hair fibres, with the *AB* genotype lambs having a lower CVFD.

## 4. Discussion

This study identified an ovine gene that appears to be a second member of the KAP22 gene family. Two variants were observed, and upon sequence alignment they were highly similar to caprine *KRTAP22-2* sequences [17]. They were most like the caprine variant *C*, which was present at a low frequency in the Liaoning cashmere goats. In contrast to goats, no gene associations were observed with variation in key fibre traits for the Chinese Tan lambs studied. This suggests potential differences in the function of *KRTAP22-2* in the two closely related species, albeit neither study investigated associations in large numbers of animals or across more than a single breed. 

The lower number of ovine *KRTAP22-2* sequence variants may reflect the population sampling approach (differences in the number of individuals tested and how representative they are of the species), an underlying difference between species, or both these things. Other *KRTAP* genes have also been revealed to be more diverse in goats than sheep. For example, eight variants of *KRTAP13-3* have been described in Liaoning cashmere goats [19], compared to only five variants of the corresponding gene in Romney-cross sheep [20]. In contrast, more variants of *KRTAP1-2* have been reported in sheep, with eleven variants described in 383 Merino × Southdown-cross lambs [21], versus six variants in 359 Longdong cashmere goats [22]. For some genes, comparable levels of diversity have been observed between the two species, such as with *KRTAP27-1* [23,24]. In these studies, it must be acknowledged that only small numbers of sheep and goats have been investigated, and they have typically been selected from a few breeds. In some cases they have been sourced from research flocks or a single farm, where inbreeding may have affected population diversity. Accordingly, drawing conclusions about the extent and impact of the sequence variation revealed may be premature.

Given the observed difference in allelic diversity, several factors could explain it. For example, if *KRTAP22-2* is expressed in sheep (noting that no transcripts were identified in the ovine EST databases), the reduced allelic diversity may reflect functional constraints in that species. Alternatively, the gene may not be expressed at all, may be expressed at very low levels, or only at only a particular stage of follicle development. In this respect, while Chen et al. [17] reported the expression of *KRTAP22-2* in goat hair follicles using an in situ hybridization approach, caution is always needed when interpreting these results, as the stringency of hybridization washes may not have been sufficiently high to eliminate non-specific binding to closely related genes, such as *KRTAP22-1*.

Further confirmation of the extent and location of *KRTAP22-2* expression is therefore needed in both sheep and goats. This might include the use of high-stringency hybridisation in situ and mRNA reverse transcription PCR with gene-specific primers and sequencing.

From a genomic perspective, ovine *KRTAP22-2* is located within a cluster of *KRTAP* genes on chromosome 1, several of which have been demonstrated to have associations with variations in wool traits. For example, *KRTAP20-2*, located approximately 25 kb on one side of the putative *KRTAP22-2* sequence, has been linked to MFC in Merino x Southdown-cross sheep [25], while *KRTAP6-1*, approximately 35 kb on the other side, is associated with MFD in Peppin Merino sheep, Merino × Southdown-cross sheep, New Zealand Romney sheep, Sandyno sheep, and Nilagiri sheep [9]. Given this proximity, linkage might be expected to produce similar association, but the presence of different associations with nearby genes suggests that any associations are due to individual gene effects rather than by linkage. Accordingly, the lack of association observed for ovine *KRTAP22-2* in this study possibly reflects a genuine absence of effect on the wool traits examined. Alternatively, if the gene is expressed in sheep it is possible that the low frequency of the *BB* genotype limited our ability to assess its effect, or that it may influence other traits not examined in this study. Further research involving larger populations with more *BB* genotypes and more trait measurements will therefore be necessary to clarify the role of ovine *KRTAP22-2* in wool fibre characteristics.

The sequence comparison supports the idea that the two ovine *KRTAP22-2* variants may represent an ancestral form of the gene. This is due to their high similarity to caprine variant *C*, which differs markedly from other caprine variants and occurs at a low frequency. This could suggest these sequences predate the emergence of the more divergent goat variants that confer some advantage. These interpretations should however be made with caution, as the analysis did not include all genotypes and only a limited number of fibre traits were examined in very few sheep or goats.

Together, these findings suggest potential species-specific differences in the evolution and regulation of *KRTAP22-2* and highlight the need for targeted expression analyses and further association studies to clarify if it has a role in wool fibre development.

## 5. Conclusions

This study confirms the presence of *KRTAP22-2* as a second member of the KAP22 family in sheep. Although genetic variation was identified, it was limited to two closely related alleles that are similar to a rare goat variant. No association was found between the *AA* and *AB* genotypes and MFD, CVFD, FDSD, and MFC, although the effect of the *BB* genotype was not effectively tested due to its low frequency in the sheep studied. The absence of *KRTAP22-2* transcripts in ovine EST databases suggest the gene is not expressed. Together these findings indicate that ovine *KRTAP22-2* may be functionally different to its goat counterpart.

## Figures and Tables

**Table 1:** Association of common *KRTAP22-2* genotypes with four fibre diameter-related measurements from Chinese Tan sheep.

Fibre Type	Fibre Trait ^1^	Mean ± SE ^2^		*p*
		*AA* (*n* = 173)	*AB* (*n* = 48)	
	MFD (µm)	16.6 ± 0.18	16.6 ± 0.23	0.934
Fine Wool	FDSD (µm)	4.1 ± 0.12	4.1 ± 0.15	0.638
	CVFD (%)	24.8 ± 0.56	24.4 ± 0.71	0.539
	MFC (°/mm)	63.4 ± 1.22	65.1 ± 1.56	0.294
	MFD (µm)	29.4 ± 0.38	30.1 ± 0.48	0.196
Heterotypic Hairs	FDSD (µm)	8.3 ± 0.17	8.2 ± 0.22	0.427
	CVFD (%)	28.3 ± 0.50	27.1 ± 0.64	0.074
	MFC (°/mm)	46.7 ± 0.79	45.7 ± 1.01	0.390

^1^ MFD—mean fibre diameter; FDSD—fibre diameter standard deviation; CVFD—coefficient of variation in fibre diameter; MFC—mean fibre curvature. ^2^ Predicted means and standard errors of those means derived from GLMs.

## Data Availability

The raw data supporting the conclusion of this article will be make available by the authors on request.
